# Integrative cancer care: crossing communication barriers

**DOI:** 10.18632/oncotarget.21890

**Published:** 2017-10-19

**Authors:** Eran Ben-Arye, Noah Samuels

**Affiliations:** Eran Ben-Arye: Integrative Oncology Program, The Oncology Service, Lin and Carmel Medical Centers, Clalit Health Services, Affiliated with Rappaport Faculty of Medicine, Technion-Israel Institute of Technology, Haifa, Israel

**Keywords:** doctor-patient communication, palliative care, integrative medicine

Working as a clinician and a researcher in the field of oncology often requires the use of interdisciplinary communication skills. Such skills are necessary in order to overcome the professional and personal barriers which frequently separate patients and their health care practitioners, as well as professionals from clinical and basic science disciplines. Despite the shared goal of healing and an ethical commitment to patients, the daily practice of medicine can present significant challenges when trying to grasp the bigger picture, where the separation between self and non-self, “*myself and the other*”, may be more a process of individual disciplines rather than one of synergy.

In an article recently published in the *Journal of Clinical Oncology*, we tell the story of Jacob, a 90-year-old patient diagnosed with advanced non-small cell lung cancer, who was referred by his oncologist to our integrative oncology service. The service provides patients with a complementary and integrative medicine (CIM) consultation and treatment program, as part of the oncology service’s supportive and palliative care program [1]. During Jacob’s treatment, the CIM practitioner applied pressure to the PC-6 acupuncture point, on the anterior aspect of the distal forearm. Initially, he reported improvement in his persistent cough, as well as anxiety. At the same time, the treatment also triggered a cathartic response, with Jacob to open up and, for the first time in his life, talk about his experience as a teenager in the Auschwitz and Buchenwald concentration camps. He described the process as a “Journey through the Valley of Death”, bringing closure to the loss of his family and acceptance of his impending death. At the same time, the CIM team experienced an empowering, post-traumatic process of growth.

The interaction between patients and their CIM practitioners is not limited to acupuncture or other CIM modalities, but an integral part of any healing process. At times, doctor-patient communication may include the rare transformative gesture, which can enable the crossing of intra-disciplinary borders. In Jacob’s case, the integration of complementary medicine in the supportive care setting set in motion an important aspect of his healing process.

Complementary medicine has, until recently, been unable to enter the well-guarded “scientific fortress” of conventional medical care [2]. The integration of CIM in supportive and palliative cancer care reflects a multi-disciplinary process taking place over the last past two decades in leading oncology centers throughout the US and worldwide [3]. The integrative oncology setting provides a unique opportunity to advance communication between patients and their oncology health care professionals [4]. Communication is an integral aspect of CIM, enabling its practitioners to provide individualized and patient-directed treatments, which are adapted at each encounter according to address the dynamic of symptoms and concerns. Patients first meet with an integrative physician (IP), a conventional doctor with extensive training and experience in CIM-related fields of practice. The IP and patient co-design a CIM treatment plan, addressing symptoms and concerns based on the published research regarding effectiveness and safety.

This integrative process has produced clinical practice guidelines for the use of CIM in supportive cancer care [5]. It has also created a multi-disciplinary, “bedside-to-bench” approach, in which basic-science and clinical research work together to provide evidence-based guidance on the effective and safe use of herbal remedies during chemotherapy and other oncology treatments [6]. In a recently published article, oncology and palliative care researchers from 16 countries in the Middle-East worked together to identify potentially harmful herb-drug interactions in their integrative oncology settings. The collaboration demonstrated that in spite of the hostility and mistrust between their home countries, these clinicians were able to advance their shared goal of integrative medicine, in a region where traditional medicine plays a central role in patients’ health belief models [7]. The role of CIM as a facilitator of collaboration should inspire further other collaborative efforts, enabling health care professionals and researchers to gain a better understanding of the integrative process. This, in turn, will open new opportunities within which we can all reveal our hidden gardens, waiting to be discovered.
